# A novel splicing mutation of the *FRMD7* gene in a Chinese family with X-linked congenital nystagmus

**Published:** 2012-01-13

**Authors:** Ying Hu, Jing Shen, Shuihua Zhang, Tao Yang, Shangzhi Huang, Huiping Yuan

**Affiliations:** 1 Department of Ophthalmology, the 2nd Affiliated Hospital of Harbin Medical University, Harbin, China; 2 Department of Medical Genetics, Institute of Basic Medical Sciences, Chinese Academy of Medical Sciences & Peking Union Medical College, WHO Collaborating Centre for Community Control of Hereditary Diseases, Beijing, China

## Abstract

**Purpose:**

To identify a potential pathogenic mutation in a four-generation Chinese family with X-linked congenital nystagmus (XLCN).

**Methods:**

Routine clinical examination and ophthalmic evaluation were performed on normal controls, two patients and two healthy members of the family. Genomic DNA was prepared from the peripheral blood of members of the family and from 50 normal controls. All coding exons and the intronic boundaries of the four-point-one (4.1), ezrin, radixin, moesin (FERM) domain-containing 7 (*FRMD7*) gene were amplified using polymerase chain reaction (PCR) followed by direct sequencing.

**Results:**

A previously unreported splicing mutation, c.163–1 G→T transversion (c.163–1 G>T), was detected preceding exon3 of *FRMD7* in the patients but not in the unaffected family members and 50 unrelated healthy individuals.

**Conclusions:**

We identified a novel mutation (c.163–1 G→T) of *FRMD7* in this Chinese family with XLCN. Our finding is the first report related to c.163–1 G→T mutation in *FRMD7*. The result expands the mutation spectrum of *FRMD7* in association with congenital nystagmus.

## Introduction

Nystagmus is a disease characterized by rapid involuntary movements of the eyes. This condition can affect one or both eyes. The oscillations may be vertical, horizontal, rotary, or mixed. Congenital nystagmus (CN) occurs at birth or within the first few months of life without sensory defect. The prevalence of CN is 1/20,000 and the etiology of CN is not clear. Congenital nystagmus is different from “sensory defect nystagmus” in that the former is a primary defect while the latter is a result of other ocular diseases, such as albinism, achromatopsia, Leber congenital amaurosis, congenital cataract, and anterior segment dysgenesis [1,2].

Congenital nystagmus is an inherited disease. The inheritance pattern is heterogeneous, but the most common form is X-linked. So far, three loci for X-Linked congenital nystagmus (XLCN, OMIM 310700, 300814, and 300589) have been mapped to chromosomes Xq26–27 [3], Xp22 [4], and Xp11.3–11.4 [5]. There are two genes that have been identified as disease-causing genes for XLCN. These include the G protein-coupled receptor 143 gene (*GPR143*) at Xp22 [4] and the four-point-one (4.1), ezrin, radixin, moesin (FERM) domain-containing 7 gene (*FRMD7*) at Xq26–27 [6]. The mutations in *GPR143* have been reported to cause ocular albinism type 1 (OA1). In other studies, mutations in *FRMD7* were identified in families that were affected with X-linked congenital nystagmus [6-15].

In this study, we performed mutation analysis in *FRMD7* of a four-generation Chinese family affected with XLCN. A novel splicing mutation (c.163–1 G→T) in *FRMD7* was detected in the patients but not in any of the healthy family members or in the 50 healthy controls. This finding suggests that the splice site mutation is responsible for the disease in the family.

## Methods

### Patients and clinical investigations

A four-generation Han Chinese family with CN was recruited from the Second Affiliated Hospital of Harbin Medical University, Harbin, China. Two patients (III:7 and IV:9) and two healthy members (III:1 and III:5) from this family participated in the study. In addition, 50 subjects that have no diagnostic features of CN were recruited from the Han Chinese population to serve as normal controls. All participants underwent routine clinical examination and ophthalmic evaluation, including best-corrected visual acuity in each eye, slit lamp biomicroscopy, ophthalmoscopy, ocular motor examination for strabismus, binocular sensorial testing of fusion and stereopsis, and the examination of the optic nerve. After informed consent, 5 ml of peripheral blood samples was collected from the participants in an ethylenediamine tetraacetic acid (EDTA)-coated Vacutainer (BD, San Jose, CA). Genomic DNA was extracted using the TIANamp Blood DNA Kit (Tiangen Biltech Co., Ltd., Beijing, China). This study was approved by the Second Affiliated Hospital, Harbin Medical University Ethics Committee of Harbin, China.

### Mutation analysis

All coding exons and exon-intron junctions of *FRMD7* (NG_012347 and NM_194277) were amplified through polymerase chain reaction (PCR) with the primers listed in Table1. Primers were designed using Primer3plus and checked for specificity using Basic Local Alignment Search Tool (BLAST). One hundred to 500 ng of genomic DNA was amplified in a 50 μl reaction volume containing the following: 50 μM dNTPs, 0.2 μM of each primer, 2U Taq DNA polymerase, and 1× PCR reaction buffer containing 1.5 mM MgCl_2_. The PCR cycling profile was as follows: denaturation at 95 °C for five min, 32 cycles at 94 °C for 30 s, annealing at the indicated temperature (Table 1) for 30 s, 72 °C for 45 s, and a final extension at 72 °C for 7 min. The PCR products were visualized on a 1.5% agarose gel containing ethedium bromide. Sequence analysis was performed with an ABI PRISM 3730XL Genetic Analyzer (Applied Biosystems, Foster City, CA). The results were analyzed using Chromas (version 1.0) software and then compared to the reference sequences of *FRMD7* using Vector NTI 10.3.0 (Invitrogen, Grand Island, NY). The result of the splice sites was predicted using Human Splicing Finder (HSF) Version 2.4.1 software. We used the Maximum Consensus value to select the reliable Branch Point position.

**Table 1:** Primers and PCR Conditions for *FRMD7*.

**Exon**	**Forward primer(5′-3′)**	**Reverse primer(5′-3′)**	**Annealing temperature**	**Product size (bp)**
1	CCTTGGGTGTGCATTACTTC	TTTGCTATTGTTGTCCCTTGAG	57 °C	459
2	CGTGCTGCAGTATCAGGTTAG	CCCTACATACCTAGCTGCAAAC	58 °C	243
3	TGACATTGCTGTTTGTTTCTACC	TCTCAAAGCCCTTTTCTCCC	60 °C	304
4	GGAGAGAGCGGTGTGTGTGTG	AGAATGGCCAGAAGCACTTG	61 °C	275
5	ATCTTGGATCTGGGAGAAGG	GCTTGGTTCTCTACCTGGC	58 °C	238
6	GAGGACAAGGGTATGCTGGA	TCAGGTTTAAGGGCTTGCTC	58 °C	281
7	GAGCTCTCAGGGTGGAAATG	ACACCCAAGTTTGAGCCAAG	58 °C	293
8	GGCGGTCCCTCTCATTAAG	AAACGATTTGCAGAAACAACC	59 °C	275
9	TTGGGATTTGAAGGTCTTTG	TCCTAAGCCTCCTGTGTTATTG	58 °C	301
10+11	TTTAGTAGCCTATTGGTTTATGGC	TCAATTCATGGAAGCATTTG	58 °C	401
12a	GAGGCTAATTTAGATCCCTCCC	CTGGCTTCATTGCAGTGGG	61 °C	620
12b	TATGTGGACAAGCCACCCC	CTTCAGCAGTTGGTGTGTTG	61 °C	613
12c	TGAATCAGAGATTCTTAAACCA	ACTGGGCCCCACACAAATAG	61 °C	649

### Prediction of mutation-induced splicing alteration

The potential effect of the c.163–1 G→T mutation on the splicing signals was predicted with the use of HSF software. Genomic DNA sequences surrounding the mutant site were input into the HSF to calculate the consensus values (CV) of potential splice sites and to search for branch points. A higher consensus value represents higher strength and more possibility for the splice site to be authentic. Altering of the splice site may cause the splicing to skip to the next authentic exon or to move to a potential splice acceptor in high strength (CV>80) with an appropriate high-strength upstream branch point (the CV>80 and located at 20–50 bp ahead of a consensus splice acceptor site).

## Results

### Clinical findings

**Figure 1:**
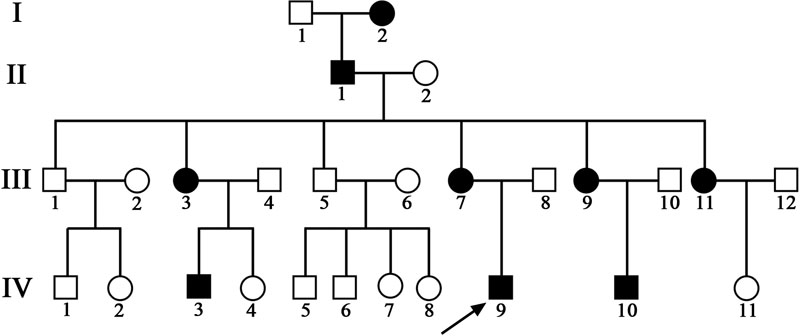
Pedigree of one Chinese family with X-linked congenital nystagmus. Peripheral blood (5 ml) was drawn from certain members (III:1, III:5, III:7, and IV:9) of the family. The squares indicate males and the circles indicate females. Black-filled symbols indicate the persons affected with CN. The proband is denoted by the black arrow.

There were nine affected individuals (five females and four males) in the four-generation Han Chinese family (Figure 1). No male to male transmission was observed. The proband (IV: 9) and his mother (III:7) had nystagmus as the first symptom. The clinical data of the participants from the family are shown in Table 2. The patients were excluded from albinism and were ascertained as X-linked congenital nystagmus based on the detailed ophthalmological examinations and their family history.

**Table 2:** The clinical finding of individuals in this study.

** **	** **	** **	** **	**Visual acuity at presentation**		** **
**Individual**	**Age**	**Sex**	**Age at onset**	**OD**	**OS**	**Clinical findings**
IV:9	7	male	since birth	20/60	20/60	rhythmical, repeated, horizontal nystagmus of both eyes
III:7	35	female	since birth	20/30	20/25	slight horizontal nystagmus of both eyes
III:1	52	male	none	20/25	20/20	normal
III:5	48	male	none	20/20	20/20	normal

### Mutation detected in *FRMD7*

**Figure 2:**
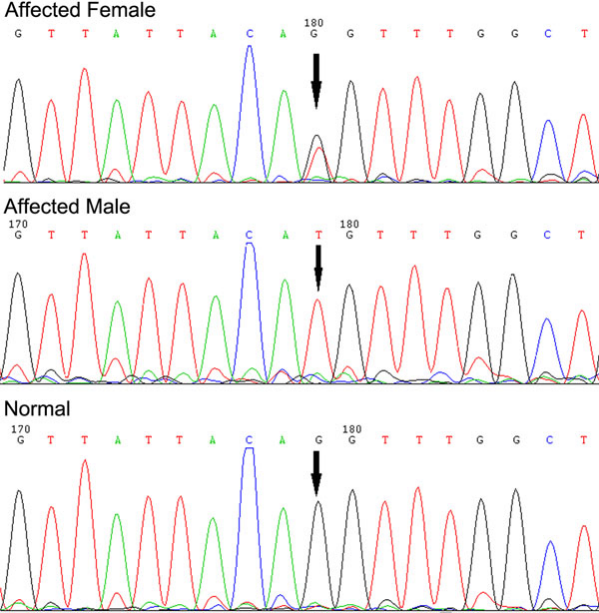
Sequence analysis of *FRMD7*. Sequencing chromatograms are from an affected female patient (top), an affected male (middle), and a normal individual (bottom), showing the c.163–1 G→T transversion.

Direct sequencing analysis detected a novel transversion, c.163–1 G→T, at the 3′-end of intron 2 of *FRMD7* in two affected individuals (Figure 2). This mutation is located within the splice junction consensus sequences and is found to be abolishing the corresponding splice acceptor site. This splice-site mutation, c.163–1 G→T, was not found in the unaffected members of the family or in the 50 unrelated controls.

### The potential effects of c.163–1G→T mutation on the splicing signal

**Figure 3:**
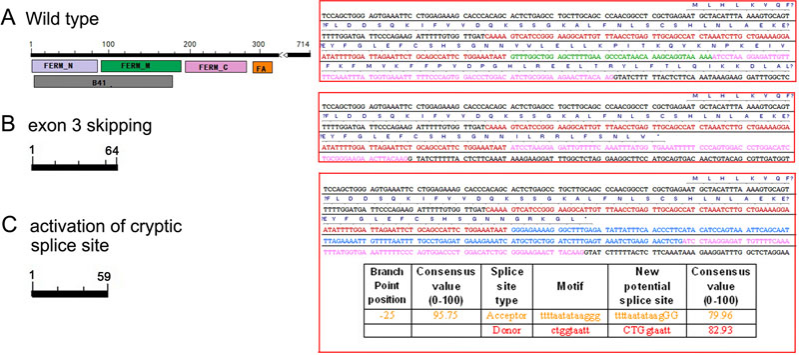
Schematic diagram showing a possible consequence from the c.163–1G→T transversion in *FRMD7*. **A**: Schematic diagram of wild type *FRMD7*. **B**: The mutant destroys the original splicing acceptor on the 3′ side of intron 2, and thus causes exon 3 (bases in green font) skipping and creates a truncated protein of 64 amino acids. **C**: Another altered splicing introduces a new stop codon and creates a truncated protein of 59 amino acids.

Sequences that surround the c.163–1 G→T site were calculated in HSF to find the cryptic splice sites by their consensus values. The consensus values indicate the strength that make the site a potential splice site. In the wild-type sequence (Figure 3A), the authentic splice acceptor at the c.163–1 site has a consensus value of 89.18 with a strong branch point (CV 87.28) of 41 bp upstream. The c.163–1G→T mutant abolishes this acceptor motif. The nearby cryptic splice sites were evaluated by their consensus value and the strength of their corresponding branch point to find the most potential splice sites. A cryptic splice acceptor (CV 79.96) in intron 3 with an appropriate high-strength branch point (CV 95.75 at −25 bp upstream) is predicted to produce a new exon with a strong downstream donor (CV 82.93; Figure 3C). Another possible splicing type induced from the c.163–1G→T mutant is generated by means of exon 3 skipping (Figure 3B). Both predicted mutant transcripts result in truncated proteins that lack the main functional domains due to mutation-induced frame shifts that lead to premature stop codon.

## Discussion

The human *FRMD7* gene consists of 12 exons spanning approximately 51 kb on chromosome Xq26-q27. It encodes a protein of 714 amino acids and shares a four-point-one, ezrin, radixin, moesin (FERM) domain at its NH_2_-terminus with band 4.1, ezrin, moesin, radixin, talin, filopodin, and merlin. The FRMD7 protein has FERM-C, FERM-M, FA, FERM-N, and B41 structural domains. The B41 domain (residues 1–192) and the FERM-C domain (residues 186–279) are highly conserved near the NH_2_-terminus. Although the function of FRMD7 is not yet clear, two other FERM domain containing proteins, pleckstrin domain protein 1 (FARP1) and FARP2, have been extensively studied. FARP1 plays an important role in the promotion of the dendritic growth of the spinal motor neuron. FARP2 has been shown to modulate the length and degree of neurite branching in developing cortical neurons.

In the present study, we report a novel G→T transversion (c.163–1G→T) identified in the splice acceptor site on the 3′side of intron 2 of *FRMD7* in an X-linked CN (XLCN) family. This splice-site mutation was not found in the unaffected members of the family or in the unrelated controls. We predicted two most possible transcripts that may have been induced by this splice-site mutation and both of them produce truncated proteins (59 amino acids and 64 amino acids, respectively) that lack the main functional domains (Figure 3B,C). The lack of functional domains was considered to seriously affect the function of these truncated proteins. The software-predicted splicing is helpful in understanding the potential effect of a splice-site mutation. So far, nine splicing mutations have been reported in *FRMD7* worldwide and all of them were linked to XLCN. Collectively, our study and published data support the theory that the splicing mutation c.163–1G→T is the pathologic mutant for XLCN in the family.

In this study, we identified a novel splice-site mutation, c.163–1G→T, in *FRMD7* of a Chinese family that has X-linked congenital nystagmus. To date, more than 40 mutations in *FRMD7* have been identified worldwide in families that have XLCN. These families come from different ethnic groups [6-12,16,17]. According to the Human Gene Mutation Database (HGMD), the mutations include missense, nonsense, splicing mutations, insertions, and deletions [18]. However, the underlying molecular mechanisms in CN are not fully understood yet. Our data expands the mutation spectrum of *FRMD7* in causing congenital nystagmus and may be helpful for understanding the molecular pathogenesis of XLCN.
